# Continuous intravenous infusion of human mesenchymal stromal cell-derived small extracellular vesicles in spinal cord injured rat modulates extracellular matrix and has greater therapeutic efficacy than multiple single injections

**DOI:** 10.1016/j.neurot.2026.e00892

**Published:** 2026-03-26

**Authors:** Masahito Nakazaki, Karen L. Lankford, Masayuki Toyoshima, Yoshiaki Tanaka, Tomokazu S. Sumida, Jeffery D. Kocsis

**Affiliations:** a Department of Neurology, Yale University School of Medicine, New Haven, CT, 06510, USA; b Center for Neuroscience and Regeneration Research, VA Connecticut Healthcare System, West Haven, CT, 06516, USA; c Department of Neural Regenerative Medicine, Institute of Regenerative Medicine, School of Medicine, Sapporo Medical University, Sapporo Hokkaido, 060-8556, Japan; d Division of Regenerative and Advanced Therapy, Nipro Corporation, Osaka, Osaka, 531-8510, Japan; e Maisonneuve-Rosemont Hospital Research Center (CRHMR), Department of Medicine, University of Montreal, Quebec, Canada; f Department of Neurology, Yale University School of Medicine, New Haven, CT, USA

**Keywords:** Human bone marrow mesenchymal stromal/stem cell-derived small extracellular vesicles, Spinal cord injury, microRNAs, Macrophages, Extracellular matrix

## Abstract

Intravenous (IV) administration of human bone marrow mesenchymal stromal/stem cell-derived small extracellular vesicles (hMSC-sEVs) improves motor recovery in spinal cord-injured rats. We previously observed that daily IV injections over three days were theapeuticaly effective, whereas a single injection with an equivalent total dose was not, indicating that a temporally dispersed regimen is crucial for efficacy. DiR-labeled hMSC-sEVs accumulated in M2 macrophages at the injury site, with excess vesicles cleared via the kidneys within 24 h. To determine whether prolonged continuous delivery can enhance therapeutic outcomes, we compared motor recovery in SCI rats receiving hMSC-sEVs via daily IV injections versus continuous infusion of the same quantity of hMSC-sEVs over 3 or 6 days via osmotic pumps. Continuous intra-jugular IV infusion using an osmotic pump over three days accelerated the onset of motor recovery compared to daily injections. Extending the infusion to six days further enhanced recovery despite the same total dose. Molecular analyses revealed that hMSC-sEVs are enriched in microRNAs targeting fibrosis pathways relative to control serum sEVs. *In vitro* uptake of hMSC-sEVs by M2 macrophages significantly suppressed the expression of genes associated with extracellular matrix production. Furthermore, MSC-sEV-treated animals showed reductions in fibronectin and collagen 1 and 5 proteins at the lesion site, compared to PBS treated rats. These findings suggest that prolonged continuous infusion of hMSC-sEVs results in greater motor function recovery than daily injections of the same amount, potentially by modulating macrophage-mediated extracellular matrix deposition.

## Graphical abstract

**Figure:**
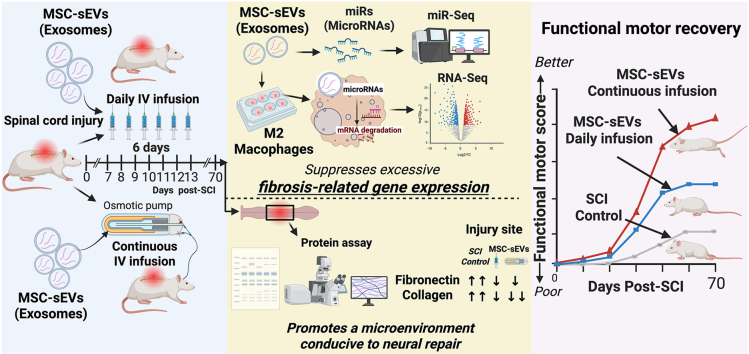

## Introduction

Spinal cord injury (SCI) induces a complex series of spatial-temporal changes in cellular composition, gene expression and extracellular matrix (ECM) deposition in and surrounding the injury site [[1], [2], [3], [4]]. The injury leads to activation of resident microglia [5] and infiltration of macrophages [6], contributing to a protracted local inflammatory response with disruption of the blood-spinal cord barrier [[7], [8], [9], [10]]. The resulting scar formation and associated collagen deposition contributes to the inhibition of axonal regeneration [11]. Over time, proinflammatory M1 macrophages/microglia, which predominate in the early lesion shift to anti-inflammatory M2 macrophages. M2 macrophages remove cell debris, secrete extracellular matrix components which influence astrocytic differentiation and cell migration, thus providing for an environment more conducive to axonal regeneration [12]. However, when ECM production, especially fibronectin and collagen I, becomes excessive, the mature fibrotic scar once again forms an inhibitory barrier [13,14]. Balanced ECM remodeling, rather than its over-accumulation, is therefore critical for successful axonal regeneration.

Mesenchymal stem/stromal cells (MSCs) from cultured bone marrow have been shown to have therapeutic effects in rat models of SCI when administered intravenously [7,[15], [16], [17]]. However, IV-delivered MSCs do not significantly target the injury site but instead aggregate in the lungs where they remain for a few days [7]. This observation suggests that MSCs exert their effects indirectly, likely via soluble or vesicle-associated factors released into the circulation. Previous studies have shown that small extracellular vesicles (sEVs) or exosome-enriched fractions, isolated from MSCs (MSC-sEVs) can reproduce many of the parent cells’ therapeutic effects in various models of injury and disease [[18], [19], [20], [21], [22], [23], [24], [25], [26], [27], [28], [29], [30], [31], [32]] including SCI [8,9,33].

Exosomes are operationally defined as a subtype of sEVs ranging from ∼30 to 150 nm in diameter, originating from the endosomal pathway and found in virtually all biological fluids [34,35]. The lipid bilayers of exosomes are highly enriched in sphingolipids and tetraspanins and are uniquely stable to detergents and low pH [36,37]. Exosomes carry complex cargoes consisting of proteins, mRNAs and microRNAs (miRs) which differ for different cells as well as the same cell types exposed to different environmental conditions. Exosomes are believed to alter the function of target cells by modulation of gene expression in cells that take up their molecular cargoes. In particular, specific miRs present in MSC-sEVs have been proposed to be key mediators of their therapeutic actions in many different injury and disease models [[19], [20], [21], [22], [23], [24], [25], [26], [27], [28], [29], [30], [31], [32]]. The specific mechanism of action of MSC-sEVs in promoting functional recovery after contusive SCI remains unclear.

Although the term “MSC-derived exosomes” was frequently used in earlier literature, isolating pure exosome populations is technically challenging due to size and marker overlap with other vesicle subtypes [38]. In line with the MISEV2023 guidelines, we therefore use the terms sEVs here to refer to nanoparticle samples isolated using the classic differential centrifugation technique for exosomes. The vesicle isolates in this study contained an abundance of vesicles smaller than 200 nm in diameter and were enriched in classical exosome-associated markers (e.g., CD63, CD9, Alix). Importantly, unlike MSCs themselves, systemically administered MSC-sEVs selectively accumulate at the SCI lesion site, where they are preferentially internalized by CD206^+^ M2 macrophages [39].

Although several investigators have shown that MSC-sEVs can enhance functional recovery in rat models of SCI, dosing, timing and methods of delivery have varied considerably [8,[40], [41], [42], [43], [44], [45], [46]]. We previously reported that IV delivery of a low dose of MSC-sEVs in three daily injections resulted in significant improvement in rat locomotor function after severe contusive SCI, whereas the same total quantity of sEVs delivered in a single dose did not [8]. Furthermore, tracing of labeled MSC-sEVs showed that IV infused MSC-sEVs were rapidly cleared via the kidneys. These findings suggest that MSCs trapped in the lungs after IV infusion exert their therapeutic effects in SCI by releasing MSC-sEVs into the circulation over time which then travel to the SCI site [7]. Indeed, we demonstrated that when membrane labeled hMSCs are intravenously delivered to SCI rats, labeled hMSC-sEVs are found in the lesion site confirming this proposal (Nakazaki et al., 2021). In the present study we test the hypothesis that prolonged continuous IV infusion of hMSC-sEVs further increases the therapeutic efficacy of hMSC-sEVs compared to repeated daily injections and modulates the extracellular matrix environment.

In this study we evaluated the relative efficacy of continuous infusion of hMSC-sEVs in SCI rats versus daily dosing over 3 days or 6 days, beginning at 1-week post-SCI. Continuous infusion of hMSC-sEVs accelerated functional recovery compared to daily injections and continuous infusion over 6 days resulted in significantly increased locomotor recovery compared to daily injections of the same total dose of sEVs. We also evaluated the miR contents of hMSC-sEVs and mRNA changes in macrophages which took up hMSC-sEVs *in vitro* and measured levels of key proteins within SCI lesions predicted from the *in vitro* analysis as targets of MSC-sEVs miRs. These data are consistent with hMSC-sEVs altering macrophage expression of collagens and fibronectin, thus changing the extracellular matrix composition in the lesioned area. Taken together these findings indicate that hMSC-sEVs have greater therapeutically efficacious when delivered by continuous infusion over several days than bolus injections and that they may act, at least in part, by altering the local extracellular matrix composition. These results have important implications for delivery protocols of MSC-sEVs to obtain maximal therapeutic benefit and suggest the importance of modulation of fibrosis pathways in SCI.

## Materials and methods

### Contusive SCI model and treatment groups

All animal experiments were carried out in accordance with National Institutes of Health guidelines for the care and use of laboratory animals and the VA Connecticut Healthcare System Institutional Animal Care and Use Committee (IACUC) approved all animal protocols. Human bone marrow-derived mesenchymal stem cells (hMSCs) were obtained from Lonza Walkersville Inc. (Walkersville, MD, USA). Human serum was purchased from CGT Global (Boston, MA, USA).

Contusive spinal cord injuries were performed on adult male Sprague–Dawley rats (185–215 g) as described previously [8,33]. Briefly, dorsal laminectomies (T9) were performed under isoflurane gas anesthesia, followed immediately by a delivery of 22.5 N impact (equal to 225 kdyn) with a 2.5 mm tip using the Infinite Horizon (IH) impactor (Precision Systems & Instrumentation, Lexington, KY, USA). Appropriate post-operative care, including twice-daily bladder expression for up to 7 days, antibiotic treatment (enrofloxacin 0.05 mg/kg/day SQ), and pain relief (buprenorphine 0.05 mg/kg/day SQ) for 48 h, was provided for all animals.

One-week post-SCI, animals which meet the inclusion criteria (see below) were randomly assigned to one of 8 treatment conditions: (1) 3-day daily injections of PBS, (2) 3-day continuous infusion with PBS, (3) 3-day daily injection of hMSC-sEVs, (4) 3-day continuous infusion of hMSC-sEVs, (5) 6-day daily injections of PBS, (6) 6-day continuous infusion of PBS, (7) 6-day daily injections of hMSC-sEVs and (8) 6-day continuous infusion of hMSC-sEVs. Daily injection treatment groups received 100 μl PBS or 100 μl PBS with 1.53 μg hMSC-sEVs per dose or a total of 4.6 μg hMSC-sEVs protein (approximately 2.5 × 10^9^-hMSC-sEVs,as estimated by protein assay and nanoparticle tracking analysis) for the 3-day treatment groups and 9.2 μg hMSC-sEVs protein (approximately 5.0 × 10^9^-hMSC-sEVs) for the 6-day treatment. Continuous infusion treatment groups were implanted with an Alzet osmotic pump (1003D, DURECT Corporation, Cupertino, CA, USA) loaded with 100 μl PBS or 9.45 μg hMSC-sEVs protein in 100 μl PBS for a total delivery of 4.6 μg hMSC-sEVs protein infused over 3 days. For the 6-day continuous infusion group, the osmotic pump was replaced after 3 days. Osmotic pumps were loaded with PBS or PBS + hMSC-sEVs, primed for 6 h in PBS at 37 °C according to manufacturer's recommendations, and implanted under isoflurane gas anesthesia. Briefly, first an incision was made lateral to the trachea, the external jugular vein was exposed and elevated, the cephalic head of the vein was ligated, and a catheter was inserted in the direction of the heart and secured with sutures. Then the distal end of the catheter was attached to the osmotic pump, a subcutaneous pocket was made in the mid-scapula and the osmotic pump was inserted and secured with sutures and the incision was closed. Post operative pain relief was provided for 48 h with buprenorphine (0.05 mg/kg/day SQ). In a preliminary experiment, we confirmed that continuous delivery using Alzet osmotic pump with 4% Evans Blue in saline (2.5 mg/kg, Sigma) functions effectively in our spinal cord injury model (Supplementary Fig. 1).

### Animal allocation, assessment of functional recovery and growth trajectory

Open-field locomotor function was assessed using the Basso-Beattie-Bresnahan (BBB) scoring system [47] by a tester blinded to the treatment. Rats were scored prior to surgery, 3-days post-SCI, 1-week post-SCI and at weekly intervals thereafter until the time of sacrifice, typically 10 weeks post-SCI. Animals were included in the study only if they had BBB scores of 0 on day 3 and 0.5 on day 7 post-SCI. Animals were weighed on the day of surgery and at weekly intervals for 10 weeks [33].

A total of 349 rats were used in this study. Of these, 340 underwent contusive SCI surgery, 6 were used for bone marrow isolation for the *in vitro* macrophage experiments, and 3 non-SCI rats served as controls for Western blot analysis. Of the 340 SCI rats, 101 met the inclusion criteria. Nine animals died or were euthanized due to complications unrelated to treatment (e.g., hindlimb infections secondary to SCI-related immobility), distributed across groups as follows: 3-day PBS daily injection (n = 1); 3-day PBS pump (n = 2); 6-day daily PBS injection (n = 2); 6-day PBS pump (n = 2); 3-day hMSC-sEVs daily injection (n = 1); 6-day hMSC-sEVs daily injection (n = 1). The remaining 92 rats were analyzed for behavioral outcomes (BBB scores and body weight). The final sample sizes for behavioral analysis were: 3-day hMSC-sEVs daily injection (n = 15), 3-day hMSC-sEVs pump (n = 15), 3-day PBS daily injection (n = 8), 3-day PBS pump (n = 7), 6-day hMSC-sEVs daily injection (n = 15), 6-day hMSC-sEVs pump (n = 16), 6-day PBS daily injection (n = 8), and 6-day PBS pump (n = 8). Western blot analysis of ECM proteins at the lesion site was performed on a subset of these animals: 6-day hMSC-sEVs daily injection (n = 5), 6-day hMSC-sEVs pump (n = 5), 3-day hMSC-sEVs pump (n = 5), 6-day PBS daily injection (n = 5), 6-day PBS pump (n = 5), and non-SCI control (n = 3). For the *in vitro* macrophage experiment, bone marrow monocytes were isolated from 6 rats and cultured in 3 conditions (control, hS-sEVs, hMSC-sEVs; 2 rats per condition).

### Human mesenchymal stem cell (hMSC) culture, isolation and characterization of hMSC-sEVs and human serum sEVs (hS-sEVs)

hMSCs (Lonza, Walkersville, MD, USA, PT-2501) were cultured and expanded according to the manufacturer's instructions and our previously reported protocol [33]. Cells were maintained in Dulbecco's Modified Eagle Medium (DMEM, Thermo Fisher Scientific, Waltham, MA, USA) supplemented with 10% fetal calf serum (FCS; Thermo Fisher Scientific), 2 mM L-glutamine, 100 U/mL penicillin, and 100 μg/mL streptomycin. Cultures were incubated at 37 °C in a humidified atmosphere with 5% CO_2_, and cells were passaged upon reaching 80–90% confluence using 0.05% trypsin-EDTA (Thermo Fisher Scientific). hMSCs were expanded to passage 6 before use in sEV collection. To prepare cells for extracellular vesicle (EV) isolation, hMSCs were washed three times with phosphate-buffered saline (PBS, Thermo Fisher) to remove residual FCS-derived vesicles and cultured in serum-free DMEM for 48 h. The conditioned medium was then harvested and subjected to differential ultracentrifugation for small EV (sEV) isolation, following MISEV2023 recommendations [38] and the protocol by Théry et al., 2006 [48]. Briefly, the media were sequentially centrifuged at 300×*g* for 10 min to remove cells, followed by 2000×*g* for 20 min to remove dead cells and apoptotic bodies, and 10,000×*g* for 30 min to eliminate larger vesicles and debris. The resulting supernatant was filtered through a 0.22 μm PES membrane (Millipore, Burlington, MA, USA), then ultracentrifuged at 100,000×*g* for 70 min at 4 °C using a fixed-angle rotor (Beckman Coulter, Brea, CA, USA). The sEV pellet was washed in PBS and centrifuged again at 100,000×*g* for 70 min to reduce contaminating proteins. Final sEV pellets were resuspended in PBS and stored at −80 °C for downstream use. As a control for nonspecific effects of sEVs uptake, small EVs derived from human serum (hS-sEVs) were prepared using the same protocol. Briefly, human serum (CGT Global, Boston, MA) was diluted to 10% in DMEM and incubated under identical conditions for 48 h. The collected medium was processed using the same differential centrifugation and filtration steps as described above for hMSC-sEVs, to ensure procedural consistency between groups.

**Fig. 1:**
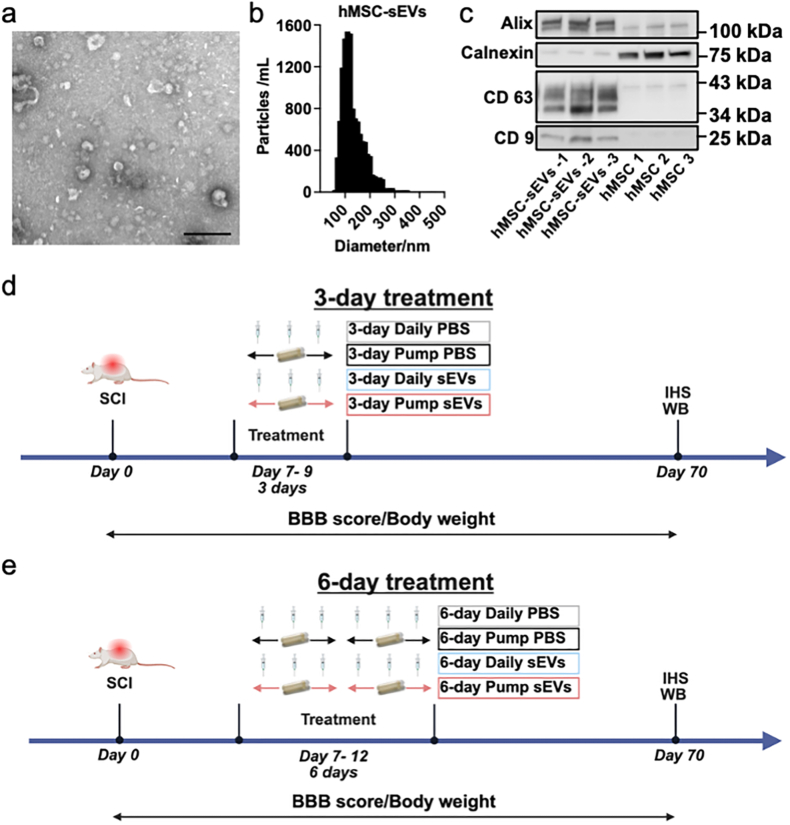
Characterization of hMSC-sEVs and experimental timelines. (a) Transmission electron micrograph of hMSC-sEVs showing typical morphology. Scale bar = 100 nm. (b) Size distribution of hMSC-sEVs, with most vesicles between 80 and 200 nm. (c) Western blot analysis of three independent hMSC-sEV preparations and their corresponding parental hMSCs. Exosomal markers Alix, CD63, and CD9 were enriched in hMSC-sEVs relative to hMSCs. The endoplasmic reticulum marker Calnexin was strongly detected in hMSC lysates but showed only minimal expression in hMSC-sEV preparations, confirming negligible contamination with intracellular organelle-derived material. (d, e) Treatment timelines for 3-day (d) and 6-day (e) protocols starting on Day 7 post-SCI. Rats received (1) Daily PBS injections, (2) PBS administered via osmotic pump, (3) daily injections of human MSC-derived sEVs(hMSC-sEVs), and (4) hMSC-sEVs administered via osmotic pump. Treatments began on Day 7 post-SCI and continued for 3 (d) or 6 (e) days. Functional recovery (BBB score) and body weight were monitored for 10 weeks; tissues were collected on Day 70 for IHC and western blot. Abbreviations: hMSC-sEVs, human mesenchymal stem cell-derived small extracellular vesicles; BBB, Basso, Beattie, and Bresnahan locomotor score; IHC, immunohistochemistry; WB, western blot; PBS, phosphate-buffered saline; SCI, spinal cord injury.

Protein levels were measured using a micro-BCA Protein Assay Kit (Thermo Fisher Scientific) and an Infinite M Plex plate reader (Tecan, San Jose, CA, USA). To analyze the composition of hMSC-sEV fractions, samples were sent to Alpha Nano Tech (Durham, NC, USA) for transmission electron microscopy and particle counting. Copper carbon Formvar grids were glow-discharged just before loading with the undiluted sample. The grids were placed on a 10 μL sample drop for 15 min, then washed twice by floating on a water drop for 30 s each. They were then negatively stained with 2% uranyl acetate by floating on a drop of stain for 30 s. After drying with Whatman paper, the grids were imaged using a Jeol 1230 electron microscope (JEOL, Peabody, MA, USA). The particle size distribution in hMSC-sEV fractions was also assessed by nanoparticle tracking analysis (NTA) using the ZetaView (Particle Metrix, Meerbusch, Germany). Western blotting was performed on three hMSC-sEV samples and their corresponding hMSCs to evaluate the enrichment of exosome protein markers, including Alix, CD63, and CD9 (Fig. 1a–c).

hMSC-sEV preparations were stored at −80 °C for up to 3 months and subjected to a single freeze-thaw cycle before injecting or loading into the osmotic pump. As described above, the osmotic pump was replaced after 3 days for the 6-day continuous infusion protocol; therefore, hMSC-sEVs were maintained within the pump reservoir for a maximum of 3 days under physiological conditions. To confirm the integrity of hMSC-sEVs under these conditions, we performed stability assays on hMSC-sEV preparations incubated at 37 °C for 3 days (Supplementary Fig. 2). NTA analysis showed that the mean particle size decreased by 11.7 ± 1.4% (n = 3) and particle concentration decreased by 19.1 ± 4.1% (n = 3) after 3 days at 37 °C, with no evidence of significant aggregation. Total protein yield, measured by micro-BCA assay, remained essentially unchanged (1.47 ± 0.88% reduction, n = 3). RNA yield, assessed using the Agilent 2100 Bioanalyzer with the RNA 6000 Pico Kit, showed a 29.3 ± 1.16% (n = 3) reduction, although the overall electropherogram profile, including the small RNA peak distribution characteristic of sEVs, was preserved (Supplementary Fig. 3). These results indicate that the structural and molecular integrity of hMSC-sEVs was largely maintained under the conditions used for osmotic pump delivery.

For *in vitro* sEV uptake experiments, hMSC-sEVs, or hS-sEVs were incubated with DilC18(7); 1,1-dioctadecyl-3,3,3′,3′-tetramethylindotricarbocyanine iodide (DiR) (Molecular Probes, Grand Island, NY, USA) for 10 min at room temperature, centrifuged at 100,000g at 4 °C for 70 min, and the pellet washed in PBS and centrifuged a second time before being resuspended in PBS and held at 4 °C until use.

### *In vitro* study of macrophage response following uptake of hMSC-sEVs

The *in vitro* macrophage experiment was conducted following the protocol we previously described [33]. Bone marrow monocytes were isolated from femurs of 6-week-old SD rats as described in Ying et al. [49] plated on 100 × 15 mm petri dishes at 5.5–7.0 × 10^6^ cells per dish (Falcon 351029) in macrophage growth media consisting of IMDM (Iscove's Modified DMEM) without HEPES (Sigma-Aldrich, St. Louis, MO, USA, I3390), with 15% heat-inactivated fetal calf serum, glutamate, sodium pyruvate, penicillin streptomycin, and 10 ng/ml colony-stimulating factor (CSF) (Sigma-Aldrich, SRP3332) and fed every 2–3 days. After 11 days, growth media was replaced with M2 macrophage induction media containing 10 ng/ml each interleukin 4 (Sigma-Aldrich, I3650) and interleukin 13 (Sigma-Aldrich, SRP4166) respectively (without CSF), adjusted to a pH of 6. Two days after transfer to M2 induction media, fresh M2 induction media containing 2 μg protein/ml DiR-labled hS-sEVs or hMSC-sEVs and 1 μl/ml of a central myelin enriched fraction [39] for 48 h prior to detachment with Macrophage Detachment Solution (Sigma-Aldrich, C41330) and then held in PBS with 2%FCS and 1% sodium pyruvate for cell sorting and RNA analysis of cells.

### RNA extraction and sequencing for sEVs

Total RNA was extracted from the isolated hMSC-sEVs and Human Serum-derived sEVs (hS-sEVs) using the miRNeasy Serum/Plasma Kit (QIAGEN, Inc., Redwood City, CA, USA) following the manufacturer's protocol. The RNA quantity was measured using a NanoDrop 2000 spectrophotometer (Thermo Fisher Scientific Inc.), and RNA integrity was assessed using an Agilent Bioanalyzer 2100 system with the RNA 6000 Pico Kit.

For small RNA sequencing, libraries were prepared using the TruSeq Small RNA Library Preparation Kit (Illumina Inc., San Diego, CA, USA) according to the manufacturer's instructions. The libraries were quantified with a Qubit Fluorometer (Thermo Fisher Scientific Inc.) and validated using the Agilent Bioanalyzer 2100 system. Sequencing was performed on an Illumina NovaSeq 6000 platform, generating 100 bp paired-end reads. Each sample was sequenced to a depth of approximately 10 million reads.

Sequencing reads were processed using miRDeep2 (version 2.0.1.3) [50], which includes read trimming, adapter removal, and alignment to the reference genome. Differential expression analysis of miRs was carried out using the DESeq2 (version 1.38.3) [51] package in R (version 4.2), with miRs considered significantly differentially expressed at an adjusted p-value <0.05. Downstream target gene prediction and pathway analysis were performed using Ingenuity Pathway Analysis (IPA) software (QIAGEN Inc.), allowing for the identification of potential regulatory networks and biological pathways influenced by the miRs present in hMSC-sEVs and hS-sEVs.

### Cell sorting, RNA extraction and sequencing from macrophages and pathway analysis to identify pathways influenced by miRs enriched in hMSC-sEVs

M2 macrophages were prepared as described above. After 48 h treatment with DiR-hMSC-sEVs or DiR-hS-sEVs, detached cells were stained with CD45-BV421 (BD Biosciences, San Jose, CA, USA) and CD11b-FITC (Bio-Rad Laboratories, Hercules, CA, USA) to confirm macrophage identity, and 7-AAD (BioLegend, San Diego, CA, USA) to exclude dead cells. DiR-positive cells were detected using APC-Cy7. Sorting was performed at the Yale Center for Genome Analysis (YCGA) using a BD FACSAria III (BD Biosciences). The gating strategy involved selecting CD45^+^ CD11b+ 7-AAD- cells as live macrophages, followed by the isolation of DiR-positive macrophages. Sorted cells were collected in RNAse-free tubes containing PBS. Details of the antibodies used are provided in Supplementary Table 1.

Total RNA was extracted from the sorted DiR-positive macrophages using the RNeasy Micro Kit (QIAGEN, Hilden, Germany) according to the manufacturer's protocol. RNA concentration and quality were assessed using a NanoDrop 2000 spectrophotometer (Thermo Fisher Scientific) and an Agilent Bioanalyzer 2100 system with the RNA 6000 Pico Kit.

For RNA sequencing, libraries were prepared from polyadenylated RNA using the SMARTer Stranded Total RNA-Seq Kit (Takara Bio Inc., Shiga, Japan). The libraries were quantified using a Qubit Fluorometer (Thermo Fisher Scientific) and assessed for quality on an Agilent Bioanalyzer 2100 system. Sequencing was conducted at the Yale Center for Genome Analysis (YCGA) on an Illumina NovaSeq 6000 platform, generating 100 bp paired-end reads. The sequencing was performed with multiplexing, allowing for multiple samples per lane, targeting approximately 10 million reads per sample.

Sequencing reads were processed and aligned to the reference genome using the STAR aligner (version 2.7.11a) [52]. After alignment, HTSeq (version 2.0.5) [53] was utilized to count the reads mapped to each gene. Differential gene expression analysis was performed using the DESeq2 (version 1.38.3) [51] package in R (version 4.2), with an adjusted p-value <0.05 considered statistically significant. IPA (QIAGEN Inc.), was employed for downstream pathway and network analysis of differentially expressed genes.

To identify pathways directly influenced by miRs within hMSC-sEVs, we conducted a detailed analysis using IPA (QIAGEN Inc.). First, the microRNA Target Filter in IPA (QIAGEN Inc.) was used to identify mRNAs that are predicted targets of miRs significantly enriched in hMSC-sEVs compared to hS-EVs. Simultaneously, RNA-seq data from macrophages treated with hMSC-sEVs were analyzed to identify mRNAs that were significantly downregulated (adjusted p-value <0.05) and showed a fold change of less than 1/4. Next, we cross-referenced the predicted microRNA targets with the downregulated mRNAs identified from the RNA-seq analysis. This allowed us to pinpoint mRNAs that were both targets of the MSC-sEV-enriched miRs and downregulated in macrophages following MSC-sEV treatment, with a stringent cutoff of fold change <1/4. Finally, the set of common mRNAs—those both predicted as targets and experimentally confirmed as downregulated—were subjected to canonical pathway analysis using IPA. This analysis enabled us to identify specific biological pathways that are likely influenced by MSC-sEV-derived miRs, shedding light on the molecular mechanisms through which hMSC-sEVs exert their effects on macrophage function.

### Immunohistochemistry

On day 70 post-SCI, rats were deeply anesthetized with sodium pentobarbital, perfused with saline, followed by 4% paraformaldehyde in 0.1 M phosphate buffer and spinal cord surrounding the lesion site was processed for standard frozen sectioning. Longitudinal sections (20 μm) taken at 1.5 mm from the centers of each block were stained with two or more of the primary antibodies directed against CD206, Collagen, and Fibronectin (Supplementary Table 1) diluted in 0.01% Triton X-100, 5% fishskin gelatin (0.1 M PBS) blocking buffer, visualized with species-specific secondary antibodies (Supplementary Table 1) counterstained with DAPI mounting media (Vectashield, Vector Laboratories, Burlingame, CA, USA) and photographed with a Nikon A1R multiphoton confocal microscope with NIS Elements software (Nikon, Tokyo, Japan).

### Western blots

For evaluation of extracellular matrix proteins at the injury site, rats were sacrificed under deep anesthesia with sodium pentobarbital. Spinal cords removed and stored at −70 °C, and total protein were purified from a homogenized 8 mm (30 mg wet weight) sections of spinal cord tissue centered around the impact sites using the RIPA Lysis and Extraction Buffer (Thermo Fisher Scientific). Protein was quantified using a BCA protein assay, and equal amounts were loaded and run on 10% SDS–polyacrylamide gels, transferred to polyvinylidene difluoride membranes, probed with appropriate primary antibodies (Supplementary Table 1) overnight at 4 °C, followed by secondary antibody staining and visualization with an enhanced chemiluminescence detection system (Thermo Fisher Scientific) using Luminescent Image Analyzer (ChemiDoc MP Imaging System, Bio-Rad) and band density analysis by ImageJ software (NIH, Bethesda, MA, USA).

### Statistical analysis

All statistical analyses were performed using GraphPad Prism software (version 9.0). Repeated-measures two-way ANOVA followed by Sidak post hoc tests were conducted for multiple comparisons of BBB scores. Continuous data were analyzed by 1-way ANOVA or the Kruskal-Wallis test. The Tukey-Kramer test or Steel-Dwass test were used to compare the subgroups. All *p* values < 0.05 were considered statistically significant. All values shown here are expressed as mean ± SEM. All methods and data were reported with consideration of guidelines provided by Animals in Research: Reporting in Vivo Experiments (ARRIVE) [54] and Minimum Information about a Spinal Cord Injury Experiment (MIASCI) [55].

## Results

### Characterization of human MSC-derived sEVs (hMSC-sEVs)

The characteristics of hMSC-sEVs samples isolated from serum-free culture media of human bone marrow–derived MSCs using the differential centrifugation method were similar to those we previously reported for sEVs derived from rat MSCs cultured under comparable conditions [8,33] and consistent with samples that were highly enriched in exosomes. Transmission electron microscopy revealed that the hMSC-sEV samples contained numerous vesicles with diameters ranging from 70 to 150 nm, which is characteristic of exosomes (Fig. 1a) and NTA analysis of the samples found that the mean particle size was 130 nm (n = 3 samples) (Fig. 1b). These size values were consistent with the standard reported measurements for human bone marrow MSC-sEVs [56]. Western blot analysis showed that hMSC-sEVs samples were highly enriched in three key exosomal surface marker proteins (CD63, CD9 and Alix), when compared to equivalent protein amounts from the cultured hMSCs from which the sEVs were isolated (Fig. 1c). The endoplasmic reticulum marker Calnexin was strongly detected in hMSC lysates but only minimally in hMSC-sEV preparations, confirming negligible intracellular contamination.

### Prolonged continuous IV delivery of hMSC-sEVs significantly improves motor recovery following severe acute SCI

Our previous studies found that infusion of a single dose of parent MSCs in SCI rats resulted in therapeutic efficacy, whereas infusion of MSC-sEVs in a single dose, in a quantity intended to mimic the amount of sEVs that could be released by the IV MSCs did not [8]. However, when the same total dose of MSC-sEVs was delivered in fractions at 3 daily injections, the MSC-sEVs replicated the therapeutic efficacy of a single MSC injections. These results implied that the efficacy of MSC-sEVs depended on their continued presence over time. We therefore investigated whether continuous infusion of hMSC-sEVs or prolonging treatment beyond 3 days could further enhance recovery by comparing locomotor recovery in SCI rats treated with PBS alone or hMSC-sEVs in PBS for 3 days (Fig. 1d) or 6 days (Fig. 1e).

**Fig. 2:**
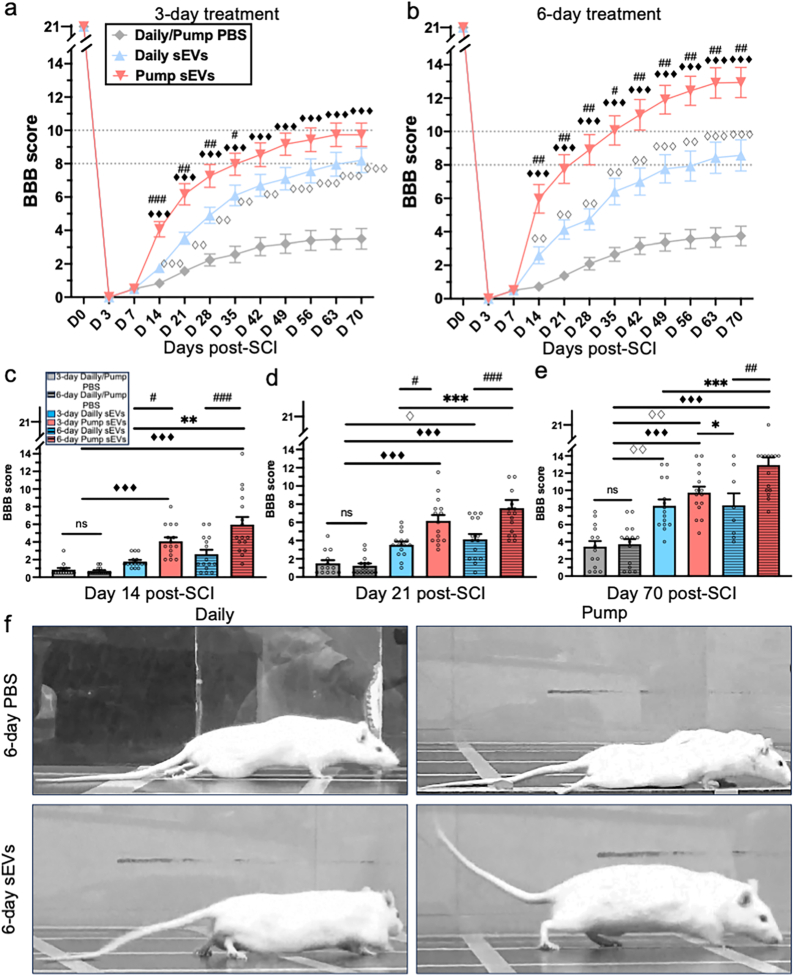
Effects of daily injections or continuous infusion of hMSC-sEVs on locomotor recovery after SCI. (a–b) Time course of BBB locomotor scores for SCI rats treated with (hMSC-sEVs) or PBS over 3 days (a) or 6 days (b). Animals received daily injections (blue symbols) or continuous pump infusion (red symbols) of hMSC-sEVs beginning at 7 days post-SCI. Grey diamonds represent PBS-treated groups (pooled data from daily injection and pump infusion conditions). Dashed lines at 8 and 10 were added to facilitate comparisons between final BBB scores for daily injection and pump infusion conditions in 3-day and 6-day treatment protocols. n = 15 for all hMSC-sEVs treatment groups, n = 7 or 8 for individual PBS treatment groups, and n = 15 for combined PBS groups (daily injection and pump infusion) with either 3-day or 6-day treatment. (c–e) Bar graphs Comparing BBB scores at: 14 days (c), 21 days (d), and 70 days (e) post-SCI. At 14 and 21 days post-SCI. Statistical significance: #p < 0.05, ##p < 0.01, ###p < 0.001 for comparisons between daily and continuous treatment groups (Daily vs. Pump); ◇◇p < 0.01, ◇◇◇p < 0.001 for comparisons between daily MSC-sEVs and PBS-treated groups; ◆◆◆p < 0.001 for comparisons between continuous MSC-sEVs and PBS-treated groups; ∗p < 0.05, ∗∗p < 0.01, ∗∗∗p < 0.001 for comparisons between other treatment groups. (f) Images showing typical body postures of locomoting rats at 70 post SCI after treatment with daily PBS injections, continuous PBS infusion, daily hMSC-sEVs injections, or continuous hMSC-sEVs infusion for 6 days. Abbreviations: SCI: Spinal Cord Injury, PBS: Phosphate-Buffered Saline, MSC: Mesenchymal Stem Cell, sEVs: Small Extracellular Vesicles, hMSC-sEVs: Human Mesenchymal Stem Cell-Derived Small Extracellular Vesicles, BBB score: Basso, Beattie, and Bresnahan locomotor rating scale.

Under the 3-day treatment protocols, animals receiving hMSC-sEVs either in daily IV injections or by continuous IV infusion via an osmotic pump, showed significantly greater functional motor recovery from day 14 to day 70 post-SCI, than animals receiving PBS via daily dosing or continuous infusion (Fig. 2a, P < 0.001 for all timepoints from 14 to 70 days post-SCI). Open field locomotor scores (BBB scores) on day 70 post-SCI were substantially increased in both the 3-day daily hMSC-sEVs injection and 3-day continuous hMSC-sEV infusion conditions, (8.2 ± 0.8, n = 15 and 9.7 ± 0.7, n = 15, respectively) compared to PBS treated with daily injection continuous infusion (3.8 ± 0.9, n = 8 and 3.3 ± 0.9, n = 7, respectively P < 0.001). Although there was no statistically significant difference between locomotor recovery of the 3-day daily hMSC-sEV injection group and the 3-day pump infusion hMSC-sEVs group at 70 days post-SCI, BBB scores were higher in the pump group than the daily injection group at earlier time points from day 14–35 post-SCI (Fig. 2a). These results indicate an accelerated time course of recovery in the 3-day hMSC-sEV pump treatment compared to daily injections, but only a trend toward improvement at the final experimental time point.

In contrast to the 3-day treatment protocols, there was a stark difference in the 6-day protocols between the efficacy of the daily injections and continuous pump infusion of hMSC-sEVs. The total hMSC-sEV dose was doubled for both the daily injection and the pump groups. Recovery in the 6-day daily injection group did not differ from the 3-day daily injection group at any time point, but the 6-day pump infusion group showed statistically significant improvement in BBB scores compared to the daily injection group at all time points from day 14–70 post-SCI. The final motor score for the 6-day hMSC-sEV treatment group was substantially higher than all other treatment conditions (Fig. 2b: 12.9 ± 0.9 for 6-day pump hMSC-sEVs compared to 9.7 ± 0.7 for the 3-day pump hMSC-sEVs, and 8.2 ± 0.7 and 8.6 ± 0.9 for 3-day and 6-day daily hMSC-sEVs injection, respectively (P < 0.001). Comparison of BBB scores for all treatment conditions at 14 (Figs. 2c), 21 (Fig. 2d) and 70 (Fig. 2e) days post-SCI indicated a substantial improvement in the 6-day pump group. At 14 days post-SCI (Fig. 2c) and 21 days post-SCI, BBB scores for both the 3-day pump hMSC-sEVs (red bar) and 6-day pump hMSC-sEVs (red striped bar) were higher than for their corresponding daily hMSC-sEV injection groups (blue bar and blue striped bar), and all hMSC-sEVs groups had greater BBB scores than the PBS treatment groups (grey bars). At the 70-day time point (Fig. 2e) the 6-day pump hMSC-sEVs treatment group showed the greatest improvement compared all other experimental groups.

The differences between locomotor scores for animals in the different treatment groups (Fig. 2a–e) were readily apparent by gross observation. Animals in control PBS and hMSC-sEVs treatment groups showed clear visual differences in recovery of both body posture (Fig. 2 f) and movement (Supplementary Video 1a-h) for the different treatment groups. At 70 days post-SCI, a representative rat which received 6-day daily PBS injections (Fig. 2f; Supplementary Video 1e) and a rat which received a continuous 6-day infusion of PBS (Fig. 2 f; Supplementary Video 1f); both demonstrated no evidence of plantar stepping, little or no evidence of hindlimb body weight support, and noticeable tail dragging. In contrast, a typical rat treated with daily injection of MSC-sEVs for 6 days showed frequent or consistent hind limb weight-supported plantar steps and occasional forelimb-hindlimb coordination with partial tail elevation (Fig. 2f, Supplementary Video 1g), while a typical rat treated with 6-day continuous MSC-sEVs infusion showed consistent hind limb weight-supported plantar stepping, as well as consistent forelimb-hindlimb coordination and normal tail elevation (Fig. 2f; Supplementary Video 1h).

Taken together, these findings suggest that although both daily injections and continuous delivery of hMSC-sEVs significantly enhances motor recovery in SCI rats, continuous infusion accelerates recovery, and extending the infusion duration to 6 days further substantially improves therapeutic outcomes. These observations underscore the importance of sustained hMSC-sEVs delivery, rather than the total dose for maximizing the therapeutic efficacy in SCI treatment.

### hMSC-sEV induced restoration of body growth is not dependent on method of delivery

**Fig. 3:**
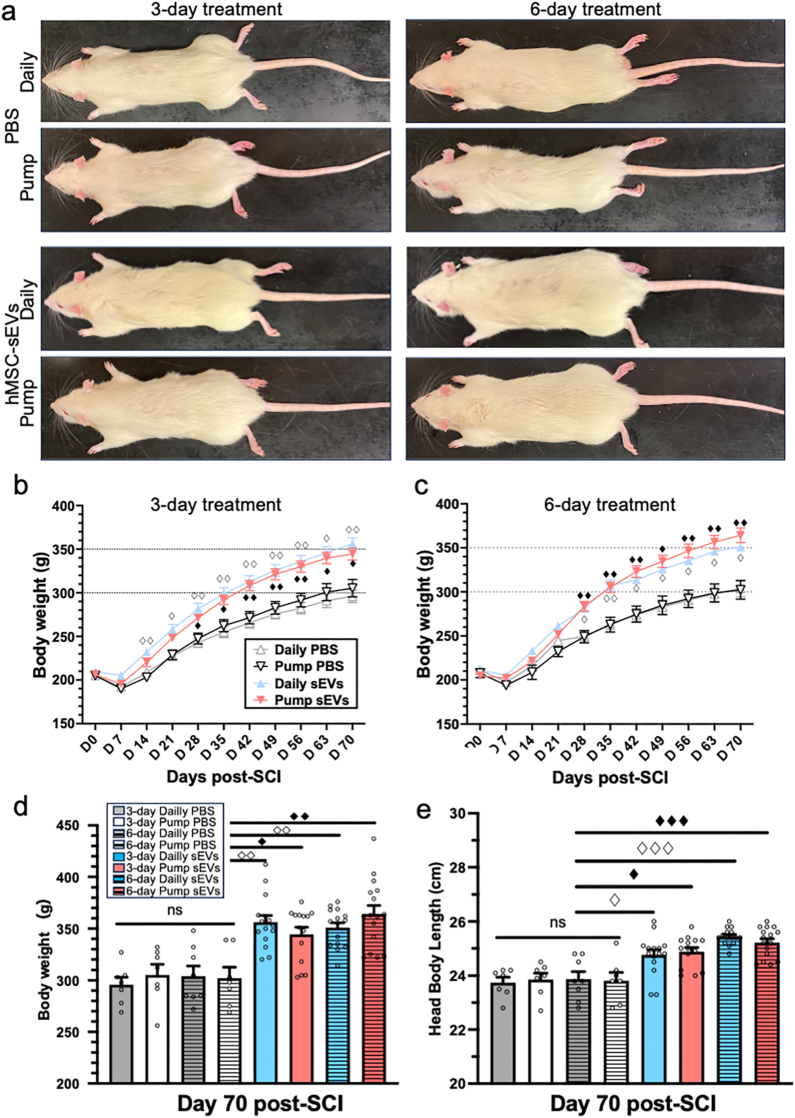
Recovery of growth trajectory after SCI. (a): Representative images showing the gross appearance rats 70 days post- SCI, following 3-day treatment or 6-day treatment with PBS vehicle alone or hMSC-sEVs in PBS. Scale bar indicates 3 cm. (i–j): Line graphs of body weight in 3-day treatment (b) and 6-day treatment (c). Dashed lines at 300 and 350 g indicate approximate weights of 3-day PBS treated and 3 day hMSC-sEVs treated groups at 70 days post SCI and are included as references to compare 3 day and 6 day treatment conditions. (d, e): Bar graphs comparing the body weight (d) and head-body lengths (e) of PBS or hMSC-sEVs treated rats 70 days post-SCI. n = 15 for all hMSC-sEVs treatment groups and 7 or 8 for individual PBS treatment groups and n = 15 for combined daily injection and pump PBS groups with 3-day or 6-day treatment. Statistical significance: ◇p < 0.05, ◇◇p < 0.01, ◇◇◇p < 0.001 for comparisons between daily MSC-sEVs and PBS-treated groups; ◆p < 0.05, ◆◆p < 0.01, ◆◆◆p < 0.001 for comparisons between continuous MSC-sEVs and PBS-treated groups. Abbreviations: SCI: Spinal Cord Injury, PBS: Phosphate-Buffered Saline, hMSC-sEVs: Human Mesenchymal Stem Cell-Derived Small Extracellular Vesicles, n: Sample Number.

In a previous study we reported that human-MSC-sEVs rapidly restored body growth, in addition to improving motor functional recovery following severe SCI in young adult rats [33]. In this study we found that both daily injection and continuous pump infusion of hMSCs for 3-days or 6-days restored growth trajectories of SCI rats with similar time course and efficacy (Fig. 3a). Rats in all PBS treatment groups appeared smaller and less well muscled than typical intact rats of their age, while rats in both the daily and continuous hMSC-sEVs groups appeared larger and more robust compared to the SCI rats in the PBS-treated groups. Weekly body weight measurements during the study period confirmed this visual impression (Fig. 3b and c). Under both the 3-day (Fig. 3b) and 6-day (Fig. 3c) dosing protocols, daily injection and pump delivery of hMSC-sEVs showed comparable acceleration of growth trajectory recovery compared to PBS treatment. Final body weights at 70-days post-SCI were higher for the hMSC-sEVs treatment groups than PBS treatment groups but body weights were not further increased by pump infusion or extending treatment time (Fig. 3d). Under the 3-day dosing protocol, the average body weights of daily and continuous PBS-treated SCI rats were 295.9 ± 7.2 g and 305.3 ± 10.0 g at 10 weeks post-SCI, respectively (Fig. 3b, d). In contrast, the mean body weights of hMSC-sEV-treated rats with daily and continuous dosing were significantly higher than those of PBS-treated animals (356.2 ± 6.6 g and 344.4 ± 7.0 g, P < 0.05, respectively) (Fig. 3b, d). However, there were no significant differences between the 3-day daily and continuous MSC-sEV-treated rats. Under the 6-day dosing protocol, a similar trend was observed. The average body weights of SCI rats treated with 6-day continuous hMSC-sEVs tended to be higher than that of rats treated with 6-day daily hMSC-sEVs, but the differences were not statistically significant (Fig. 3c and d).

Measurements of head-to-body length at 70 days post-SCI yielded comparable results as body weight data. In agreements with previous reports [33], rats treated with hMSC-sEVs had longer head-body lengths than PBS-treated rats. However, as with body weight, no differences were detected between daily and continuous dosing method nor between the 3-day and 6-day treatment protocol in the MSC-sEV treated SCI rats (Fig. 3e). Thus, unlike the effects of hMSC-sEV treatment on locomotor recovery, there were no differences between weight gain and body-to-head length for the different hMSC-sEVs treatment groups on day 70 post-SCI. All hMSC-sEVs treatment groups demonstrated increased weight gain and body-to-head length.

### microRNAs related to fibrosis pathways were enriched in the hMSC-sEVs

**Fig. 4:**
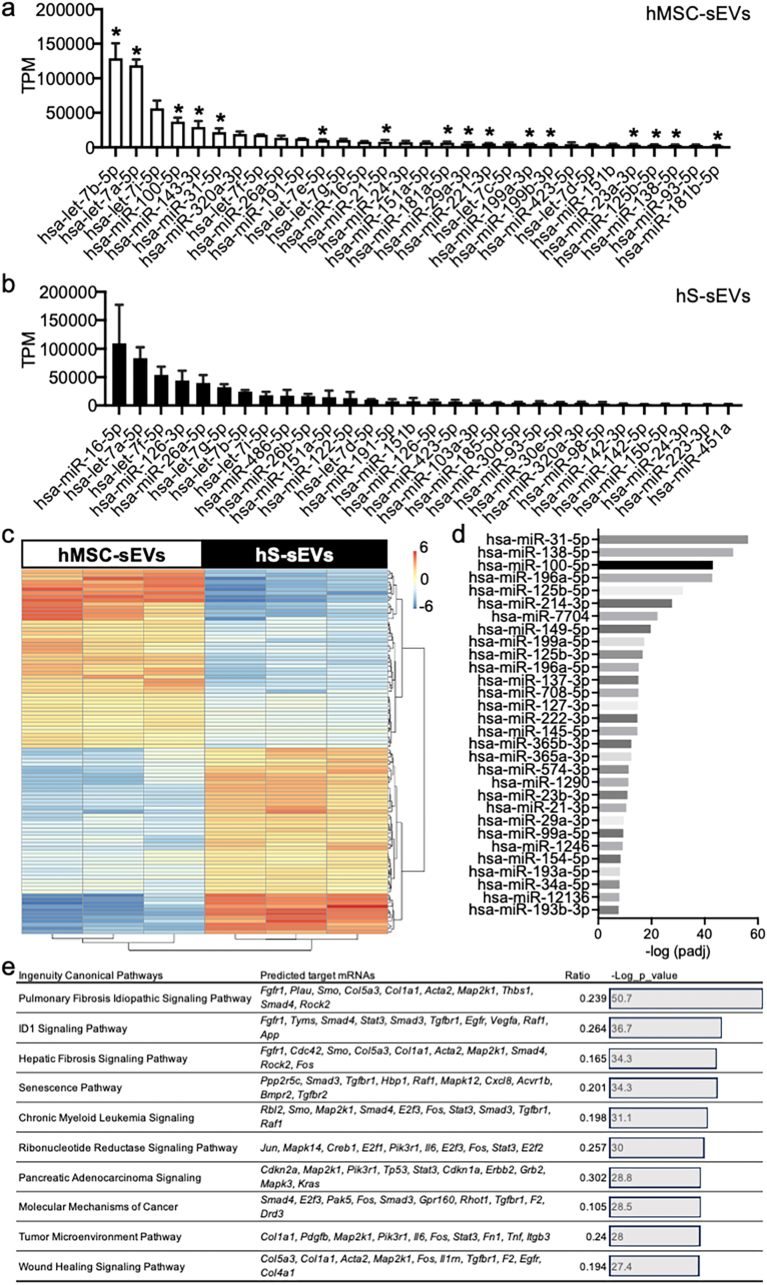
Differential expression of microRNAs in hMSC-sEVs and hS-sEVs. (a–b): Bar graphs showing the 30 most abundant microRNAs detected in 3 MSC-sEVs (a) and 3 hS-sEVs (b) samples expressed as transcripts per million (TPM). MicroRNAs significantly enriched in hMSC-sEVs are indicated by asterisks (∗, p < 0.05), compared to the hS-sEVs. (c): Heat map illustrating differentially expressed microRNAs for 3 MSC-sEVs and 3 hS-sEVs samples above, with red-yellow indicating a relative increase in expression and blues indicating a relative decrease in expression compared to the other sEVs type. (d): Graph showing the relative increase in 30 microRNAs which were significantly increased in hMSC-sEVs, compared with hS-sEVs. (e): Ingenuity Pathway Analysis (IPA) of predicted target genes of the top 30 differentially upregulated microRNAs in hMSC-sEVs. Abbreviations: hMSC-sEVs: Human Mesenchymal Stem Cell-Derived Small Extracellular Vesicles, hS-sEVs: Human Serum-Derived Small Extracellular Vesicles, TPM: Transcripts Per Million, IPA: Ingenuity Pathway Analysis.

To better understand the molecular mechanisms of action of hMSC-sEVs we investigated their cargoes, focusing on next-generation sequencing of microRNAs (miRs) and comparing them to those of control human serum sEVs (hS-sEVs). hS-sEVs were used as a control for the effects on nonspecific sEVs uptake because serum sEVs are abundant in the circulation as well as in the MSC culture media and, unlike hMSC-sEVs, pilot tests showed that EV infusion of hS-sEVs had no effect on recovery in our SCI model. This hS-sEVs control allowed us to distinguish between functionally relevant miRs selectively enriched in hMSC-sEVs from background miRs present in non-therapeutic sEVs which macrophages would be exposed to. The top 30 most abundantly expressed miRs in hMSC-sEVs and hS-sEVs are listed in Fig. 4a and b, respectively. Interestingly, some miRs were enriched in both, although their relative abundances often differed greatly between hMSC-sEVs and hS-sEVs (see Supplementary Fig. 4). For example, hsa-let-7i-5p, was the third most abundant miR in hMSC-sEVs and the eighth most abundant in hS-sEVs. Many members of the hsa-let-7 family were prevalent in both samples. The heatmap from a differential analysis between MSC-sEVs and hS-sEVs miRs showed that 119 miRs were increased (red and yellow), and 113 miRs were decreased (blue) in hMSC-sEVs compared to hS-sEVs (Fig. 4c). The top 30 most upregulated miRs in the hMSC-sEVs, compared to hS-sEVs are listed in Fig. 4d. Using Ingenuity Pathway Analysis (IPA) for these top 30 differentially upregulated miRs, we leveraged microRNA target filters, sourcing data from TarBase, miRecords, TargetScan, and the Ingenuity Knowledge Base to identify potential target genes of these miRs. This comprehensive analysis identified 914 genes targeted by these miRs (Fig. 4e). Pathway analysis revealed that these genes are significantly involved in fibrosis-related pathways, including pulmonary fibrosis idiopathic signaling, hepatic fibrosis signaling, tumor microenvironment modulation, and wound healing processes (Fig. 4e), arguing that modulation of fibrosis pathways might play a role in the observed therapeutic effects of hMSC-sEVs.

### hMSC-sEVs alter gene expression in M2 macrophages *in vitro*

**Fig. 5:**
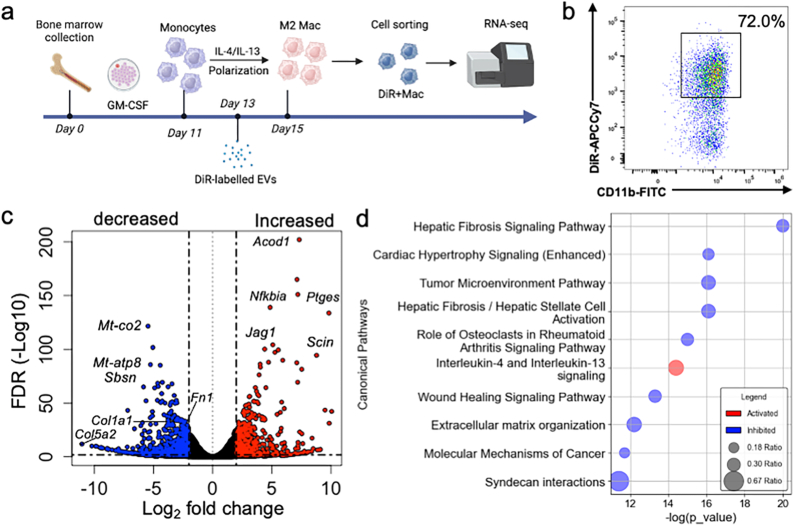
Differentially expressed genes in M2 macrophages after uptake of hMSC-sEVs versus hS-sEVs. (a) Experimental workflow (b) Fluorescence-Activated Cell Sorting (FACS)**:** FACS was performed to isolate macrophages that had taken up DiR-labeled MSC-derived extracellular vesicles (MSC-EVs)The dot plot shows DiR fluorescence (y-axis, APC-Cy7) versus CD11b expression (x-axis, FITC). The gated region represents CD11b^+^ macrophages that internalized DiR-labeled MSC-EVs. (c) Volcano plot of differentially expressed genes (DEGs) which were upregulated (red) or downregulated (blue in hMSC-sEVs treated cells, compared to hS-sEVs treated cultures. (d) Ingenuity Pathway Analysis (IPA): Pathway analysis of DEGs induced by MSC-sEV uptake in M2 macrophages identified significant modulation of several signaling pathways. Abbreviations: hMSC-sEVs: Human Mesenchymal Stem Cell-Derived Small Extracellular Vesicles, hS-sEVs: Human Serum-Derived Small Extracellular Vesicles, IL: Interleukin, DiR: 1,1′-Dioctadecyl-3,3,3′,3′-Tetramethylindotricarbocyanine Iodide, FACS: Fluorescence-Activated Cell Sorting, DEGs: Differentially Expressed Genes, IPA: Ingenuity Pathway Analysis, FITC: Fluorescein Isothiocyanate, APC-Cy7: Allophycocyanin-Cyanine7.

Although computational microRNA target prediction algorithms suggested that miRs enriched in hMSC-sEVs have the potential to modulate fibrosis-related pathways, it remained unclear whether such modulation occurs specifically within M2 macrophages. In our previous studies we found that intravenously infused MSC-sEVs were selectively taken up by CD206^+^ M2 macrophages at the lesion site, rather than by M1 macrophages or other cell types [8,33,39]. To further investigate whether hMSC-sEVs can functionally modulate fibrosis-associated pathways within these cells, we cultured M2 macrophages under conditions designed to optimize sEV uptake, exposed them to DiR-labeled hMSC-sEVs or hS-sEVs (along with myelin debris to enhance uptake) for 2 days, detached and sorted macrophages that took up DiR-labeled sEVs using fluorescence-activated cell sorting (FACS) and extracted total RNA from these DiR positive macrophages for RNA sequencing (Fig. 5a). Flow cytometry analysis confirmed that 70.8 ± 2.0% of macrophages internalized DiR-labeled hMSC-sEVs (Fig. 5b), whereas only 37.0 ± 1.0% internalized DiR-labeled hS-sEVs (n = 3 per group; P < 0.001). Principal component analysis (PCA) revealed that macrophages treated with hS-sEVs exhibited gene expression profiles that were similar to the control (CTR, non-sEV treatment) group (Supplementary Fig. 5), indicating minimal transcriptional changes in response to hS-EV treatment. However, macrophages treated with hMSC-sEVs showed a marked shift in mRNA expression, demonstrating significant changes compared to both the untreated control and hS-EV-treated groups (Supplementary Fig. 5). Analysis of differentially expressed genes (DEGs) between macrophages in the two conditions revealed significant differences in gene expression profiles between the two groups, with 1564 genes upregulated in hMSC-sEVs treated macrophages and 1445 genes downregulated in cells which took up hMSC-sEVs compared to those which took up hS-sEVs (Fig. 5c). Using the IPA software, we identified a number of signaling pathways that were significantly affected by hMSC-sEVs uptake (Fig. 5d). Notably, fibrosis and extracellular matrix-related pathways, such as the hepatic fibrosis signaling pathway, wound healing signaling pathway, and extracellular matrix organization pathways, were downregulated in activated M2 macrophages by hMSC-sEV treatment.

**Fig. 6:**
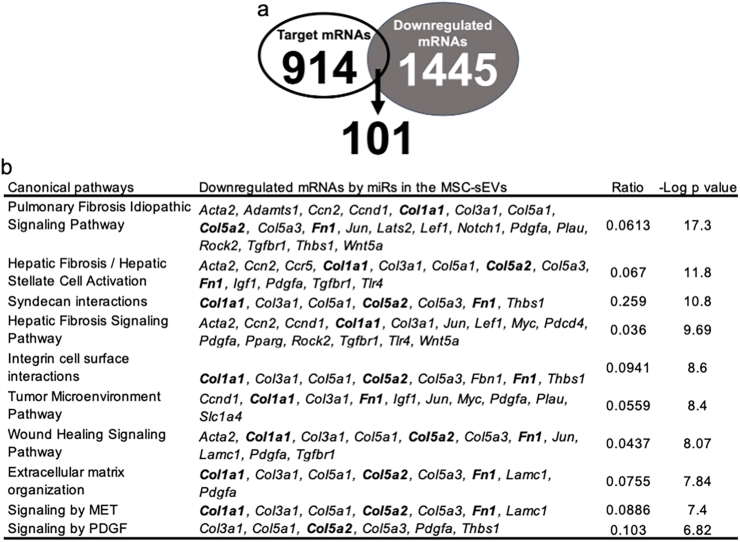
Downregulation of mRNAs in M2 macrophages treated with MSC-sEVs and associated pathways. (a) Diagram illustrating the selection of mRNAs for pathway analysis. A total of 914 mRNAs were predicted as potential targets of microRNAs in hMSC-sEVs, while 1445 mRNAs were actually observed to be downregulated in M2 macrophages treated with hMSC-sEVs compared to those treated with hS-sEVs. Among these, 101 mRNAs were both predicted targets and downregulated. (b) Pathway analysis for the 101 downregulated genes identifying key signaling pathways affected. Notably pulmonary fibrosis idiopathic signaling pathway, hepatic fibrosis signaling pathway, and wound healing signaling pathway were all strongly affected, with genes like *Col1a1, Col3a1*, *Col5a3,* and *Fn1* (bold) predicted to be downregulated through multiple pathways. Abbreviations: mRNA: Messenger Ribonucleic Acid, hMSC-sEVs: Human Mesenchymal Stem Cell-Derived Small Extracellular Vesicles, hS-sEVs: Human Serum-Derived Small Extracellular Vesicles, *Col1a1*: Collagen Type I Alpha 1 Chain, *Col3a1*: Collagen Type III Alpha 1 Chain, *Col5a3*: Collagen Type V Alpha 3 Chain, *Fn1*: Fibronectin 1.

Computational analysis predicted 914 potential gene targets of miRs that were specifically enriched in hMSC-sEVs. Further analysis revealed that 1445 mRNAs were significantly downregulated in M2 macrophages treated with hMSC-sEVs compared to the control group. To elucidate the specific mRNAs directly regulated by these miRs, we conducted a focused analysis, which identified 101 genes that were both predicted targets of hMSC-sEV miRs and were downregulated in hMSC-sEV treated M2 macrophages (Fig. 6a). Using these identified genes, we performed pathway analysis to assess the biological changes directly induced by the hMSC-sEVs uptake. The analysis revealed significant modulation of several critical signaling pathways, as illustrated in Fig. 6b. Notably, pathways related to fibrosis and extracellular matrix organization, such as the pulmonary fibrosis idiopathic signaling pathway, hepatic fibrosis signaling pathway, and wound healing signaling pathway appeared to be directly impacted by the miRs from hMSC-sEVs in the activated M2 macrophages. In particular, production of collagens (*Col1a1* and *Col5a1-3*) and fibronectin (*Fn1*), which were predicted targets of miRs in the hMSC-EVs, were downregulated by miRs in hMSC-sEVs (Fig. 6, bold). Collectively, these experiments demonstrated that hMSC-sEVs significantly modulate gene expression in M2 macrophages, downregulating key fibrosis and extracellular matrix-related pathways in a manner which would have been predicted by the miRs identified as being enriched in hMSC-sEVs. These findings suggest that modulation of fibrosis-related pathways is a potential mechanism by which hMSC-sEVs exert their therapeutic effects.

### hMSC-sEV treatment reduces fibrosis-related proteins at the lesion site in SCI rat

**Fig. 7:**
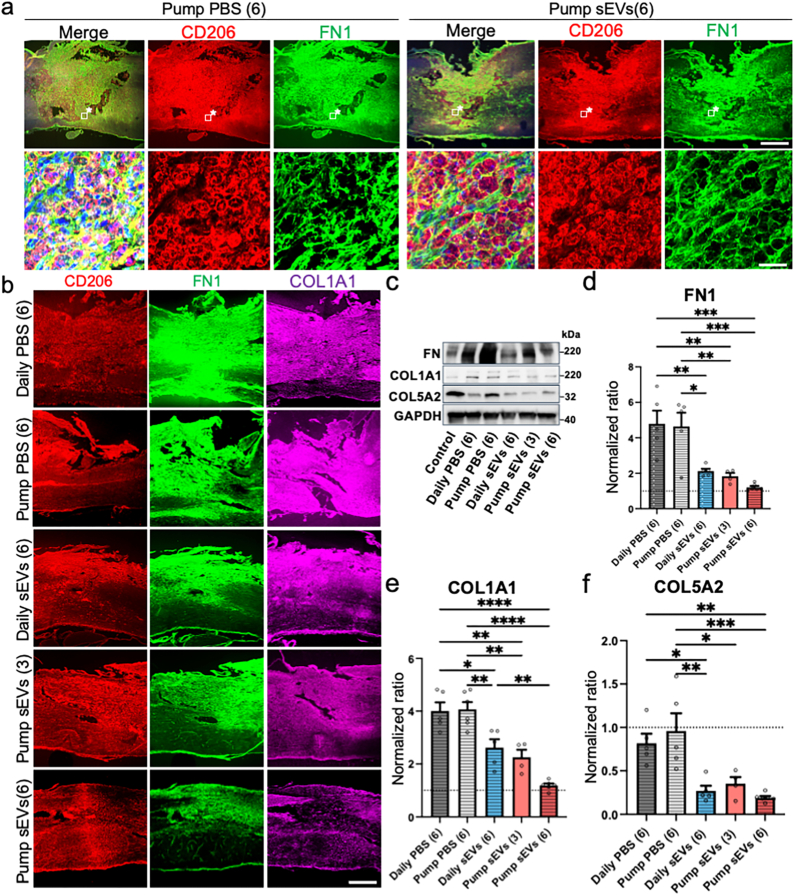
Expression of Collagen and Fibronectin at the lesion site. (a) Fluorescence micrographs showing lesion sites in representative animals treated with PBS or hMSC-sEVs at 14 days post-SCI, stained with CD206 (red) and fibronectin (green). Enlarged views of boxed areas from a and b, highlighting the co-localization of CD206 and fibronectin. (b) Fluorescence micrographs showing lesion sites in representative animals treated with PBS or hMSC-sEVs at 70 days post-SCI, stained with CD206 (red), fibronectin (green), and collagen 1 (magenta). (c) Western blot analysis of fibronectin, collagen 1, and collagen 5 levels in spinal cord segments centered around the lesion site at 70 days post-SCI, comparing PBS and hMSC-sEVs treated animals. (d–f) Quantitative analysis of western blot data for fibronectin (d), collagen 1 (e), and collagen 5 (f) normalized to control levels, showing significant reductions in extracellular matrix protein deposition in hMSC-sEV-treated groups compared to PBS-treated groups. Scale bar in lower right a = 20 μm and applies to all other high magnification images. Scale bars in upper right a and lower right b = 100 μm and apply to all low magnification images. Boxed areas marked with an asterisk (∗) in the top row indicate the regions shown at higher magnification in the bottom rowStatistical significance: ∗p < 0.05, ∗∗p < 0.01, ∗∗∗p < 0.001. ∗∗∗∗p < 0.0001. PBS: Phosphate-Buffered Saline, Abbreviations: hMSC-sEVs: Human Mesenchymal Stem Cell-Derived Small Extracellular Vesicles, SCI: Spinal Cord Injury, CD206: Cluster of Differentiation 206.

As the *in vitro* experiments suggested that hMSC-sEVs uptake by macrophages decreased production of key extracellular matrix proteins, we evaluated whether this process also occurred *in vivo* after hMSC-sEVs infusion in SCI rats. Immunohistochemical analysis of lesions at day 14 post-SCI (7 days post-treatment onset) showed significant depositions of Fibronectin 1 (FN1) (a key fibrosis-related protein) in the macrophage rich region surrounding the lesion site in the control group which received PBS continuously (Fig. 7a, left panels). At higher magnification, the Fn1 deposition could be seen to be predominantly co-localized with the M2 macrophage marker CD206, suggesting that the macrophages might be one of the key sources of FN1 production at the lesion site. Type I collagen (COL1A1), another extracellular matrix protein, showed a similar distribution pattern (data not shown). By comparison, rats that received a 6-day continuous delivery of hMSC-sEVs, showed a reduction in Fn1 deposition around M2 macrophages at the lesion site on day 14 post-SCI (Fig. 7a, right panels).

The effect of hMSC-sEVs on extracellular matrix deposition at lesion sites persisted to the end point of the study, i.e.70 days post-SCI. SCI rats that received only control PBS treatment as daily injection or continuous infusion for 6 days exhibited significant deposition of FN1 and COL1A1 around the lesion site at 70 days post-SCI, with some signals colocalized with the M2 macrophage marker CD206 (Fig. 7b). In contrast, rats treated with hMSC-sEVs showed reduced deposition of Fn1 and COL1A1 compared to the PBS-treated group. Notably, the group treated with a 6-day continuous delivery of hMSC-sEVs, which produced the highest level of functional recovery, also displayed a substantially greater reduction in these extracellular matrix proteins than the other hMSC-sEV treatment groups (Fig. 7b).

These immunohistochemical observations of FN1 and COL1A1 staining were corroborated by quantitative analysis of protein expression at the lesion site. At 70 days post-SCI, western blot analysis revealed increased band densities of finbronectin (FN1), collagen Ⅰ (COL1A1), and collagen Ⅴ (COL5A2) in all lesion conditions, compared to non-SCI control rats. However, the densities of these bands were notably reduced in all hMSC-sEV-treated conditions, compared to all PBS-treated controls (Fig. 7c). Quantification of band densities revealed that levels of FN1 (Fig. 7d), COL1A1 (Fig. 7e), and COL5A2 (Fig. 7f) were all significantly lower in all MSC-sEV-treated groups compared to all PBS-treated groups. Importantly, the 6-day continuous hMSC-sEV infusion treatment resulted in a significantly greater reduction in COL1A1 expression than the 6-day daily hMSC-sEV injection treatment receiving the same total amount of hMSC-sEV (Fig. 7e).

## Discussion

The results presented here indicate that prolonged continuous intravenous infusion of hMSC-sEVs significantly enhances the therapeutic potential of hMSC-sEVs for treatment of severe SCI when compared to daily injections. The hMSC-sEVs were administered to SCI rats beginning one week after injury either by repeated daily intravenous injections or continuous intravenous infusion via a mini-osmotic pump for three or six days. Among these methods, six days of continuous pump infusion of hMSC-sEVs resulted in markedly better functional recovery compared to other protocols, even when the same total dose of sEVs was delivered via daily injections. Molecular biological analysis of microRNA contents of hMSC-sEVs and gene expression in cultured macrophages which took up hMSC-sEVs suggested that reductions in macrophage expression of collagens and fibronectin may may be associated with hMSC-sEVs treatment. Furthermore, immunohistological staining and protein quantification at injury sites confirmed reductions in these key extracellular matrix proteins after hMSC-sEVs treatment and the degree of reduction in these proteins roughly correlated with the extent of recovery. Taken together, these findings underscore the importance of the timing of delivery of hMSC-sEVs in determining therapeutic efficacy and highlight a potential mechanism by which hMSC-sEVs may facilitate tissue repair and functional recovery following SCI by targeting M2 macrophages and modulating excessive production of fibrosis-related proteins.

The increased efficiency of hMSC-sEVs delivered by continuous infusion is consistent with our previous observations that labeled MSC-sEVs are rapidly cleared through the kidneys [39]. These observations suggest that low levels of MSC-sEVs are sufficient to induce therapeutically beneficial changes in cell functioning, but that higher doses of MSC-sEVs are required to maintain a sufficient level of circulating sEVs for several days if they are given in a single dose. Thus, a continuous infusion protocol is not only more efficacious, but makes more efficient use of perhaps a limited supply of treatment product.

Our analysis of sEVs cargoes identified several miRs that were enriched in therapeutically effective human MSC-sEVs (hMSC-sEVs) compared to therapeutically neutral human serum derived sEVs (hS-sEVs). As both types of sEVs can be taken up by macrophages under the same culture conditions, but only the hMSC-sEVs have therapeutic effects on SCI, miRs that were specifically enriched in hMSC-sEVs may be linked to their therapeutic effects. Significant differences were observed in microRNA expression profiles of hMSC-sEVs and hS-sEVs. Notably, the microRNAs miR-31–5p, miR-138–5p, and miR-100–5p were all significantly upregulated in hMSC-sEVs. Previous studies have shown that miR-31–5p plays a pivotal role in reducing the expression of pro-inflammatory factors [57], thereby mitigating the inflammatory response associated with SCI and contributing to creating a more favorable environment for tissue repair and functional recovery. Furthermore, other studies also indicate that miR-31–5p can promote neural stem cell proliferation [58] and may promote restoration of motor function after spinal cord injury. Similarly, miR-138–5p has demonstrated significant anti-inflammatory effects in SCI models, where it suppresses inflammation by targeting the NLRP3-CASPASE1 signaling pathway, which is crucial for modulating the inflammatory response in neural cells [59]. Moreover, the downregulation of miR-138–5p after SCI has been associated with the overexpression of pro-apoptotic proteins, including CASP-3, CASP-7, and BAK, exacerbating neuronal damage [60]. The significant upregulation of miR-100–5p in hMSC-sEVs also underscores its potential as a key regulator of neuroinflammation and apoptosis. By inhibiting the TLR4/NF-κB signaling pathway, miR-100–5p effectively attenuates microglial activation and neuronal cell death. Using a similar but slightly different contusive SCI model, a decrease in miR-100–5p levels was associated with upregulation of pro-inflammatory and apoptotic markers, whereas administering a chemically synthesized miR-100–5p mimic reversed these pathological changes [61]. This highlights the potential pivotal role of miR-100–5p in modulating the neuroinflammatory milieu and promoting neuroprotection following SCI.

Although several miRs enriched in hMSC-sEVs have been linked to specific pathways which could contribute to the therapeutic effects of MSC-sEVs, miRs rarely function as isolated entities. Instead, they orchestrate regulatory interactions within intricate networks, modulating biological processes through coordinated and synergistic mechanisms [62,63]. To explore the broader biological impact of the microRNA cargo in hMSC-sEVs, we utilized IPA to predict target mRNAs and identify enriched signaling pathways based on the collective set of miRs upregulated in hMSC-sEVs. The pathway analysis demonstrated that the bulk of enriched miRs were involved in a several critical regulatory pathways such as the Pulmonary Fibrosis, Idiopathic Signaling Pathway, and the Hepatic Fibrosis Signaling Pathway, supporting the hypothesis that hMSC-sEVs influence cell functions by delivering a coordinated set of miRs that target multiple signaling pathways.

*In vitro* data in this study confirmed the predictions of the pathway analysis, as genes implicated in fibrosis (predicted as targets of the enriched hMSC-sEV miRs through IPA computational analysis) were downregulated in macrophages which took up hMSC-sEVs. Specifically, key fibrosis-related genes within the Hepatic Fibrosis Signaling Pathway exhibited altered expression levels in response to hMSC-sEV uptake. These findings are consistent with the hypothesis that hMSC-sEVs alter macrophage gene expression in a manner consistent with the computational analysis. Predictions based on comprehensive computational analysis of miRs enriched in hMSC-sEVs identified 914 mRNAs as potential targets of the enriched miRs in hMSC-sEVs. In parallel, 1445 mRNAs were found to be downregulated in macrophages after uptake of hMSC-sEVs. Importantly, a total of 101 mRNAs were shared between the computationally predicted targets and the experimentally downregulated mRNAs, identifying these mRNAs as likely direct targets of MSC-sEV-derived miRs. Pathway analysis of these 101 mRNAs revealed their involvement in critical biological pathways, including the Pulmonary Fibrosis Idiopathic Signaling Pathway, Hepatic Fibrosis Signaling Pathway, and Wound Healing Signaling Pathway, among others. Many of these pathways are closely linked to the regulation of fibrosis resolution, tissue remodeling and repair, suggesting that MSC-sEVs may play a role in mitigating excessive fibrosis. Notably, fibronectin (*Fn1*), collagen I (*Col1a1*), and collagen V (*Col5a1-3*) mRNAs emerged as downregulated genes across multiple pathways targeted by MSC-sEV-derived miRs (Fig. 6b), highlighting the potential role of regulation of extracellular matrix proteins in mediating the anti-fibrotic and tissue remodeling effects of hMSC-sEVs.

Importantly, the analytical framework employed in this study was designed to capture the coordinated action of the entire set of miRs enriched in hMSC-sEVs, rather than to attribute causality to any single miR species. The convergence of multiple independent miRs on the same mRNA targets, such as Col1a1, Col5a1-3, and Fn1, provides stronger evidence for coordinated regulation than would be expected from the action of any single miR. Although functional validation through gain- or loss-of-function experiments (e.g., antagomirs, miR mimics, or knockdown approaches) would be necessary to establish direct causality for specific miR-mRNA interactions, the coordinated nature of miR-mediated regulation [62,63], argues that targeting individual miRs may not fully capture the therapeutic mechanism, which likely depends on the collective action of the miR cargo as a whole.

Consistent with the downregulation of collagens and fibronectins predicted by *in vitro* analysis of gene regulation in macrophages which took up hMSC-sEVs, the immunohistochemical and Western blot analyses of lesioned tissue revealed that treatment with hMSC-sEVs significantly reduced the expression of fibronectin (FN1), collagen I (COL1A1), and collagen V (COL5A2) in injured spinal cord tissue, compared to PBS-treated controls. These results suggest that hMSC-sEVs may suppress excessive deposition of extracellular matrix components at the lesion site, of which excessive deposition is a hallmark of pathological fibrosis.

The co-localization of CD206-positive M2 macrophages with fibronectin staining further supports the role of M2 macrophages as a key source of the extracellular matrix deposition at the lesion site. Although these observations align with the hypothesis that hMSC-sEVs deliver bioactive miRs to macrophages, which downregulate fibrosis-associated genes such as collagen and fibronectin, it is essential to recognize that extracellular matrix (ECM) components, such as fibronectin, play both positive and negative roles in tissue responses to injury. For example, fibronectin is a potent facilitator of peripheral neurite outgrowth, primarily through interactions with the beta-1 integrin heterodimers and its expression is markedly elevated in regenerating peripheral nerves, particularly at lesion sites, where it promotes Schwann cell migration and growth cone extension [64]. Similarly, type I collagen scaffolds have been demonstrated to support axonal growth and neural regeneration in SCI models [65,66]. Fibronectin, aligned with other ECM proteins, provides a structural and biochemical foundation for axonal growth and guidance, forming an essential substrate for successful neural repair. Notably, fibronectin initially exists in a soluble form but is assembled into an ECM complex by 7 days post-SCI. This matrix, while critical for initial repair, can contribute to an inhibitory environment that hinders axon regeneration if excessively accumulated [67]. Thus, while reducing excessive ECM deposition is vital for preventing pathological fibrosis, it is equally important to maintain an optimal balance of ECM components to foster axon regeneration and promote functional recovery. Balancing these dynamics is critical for the effective therapeutic application of hMSC-sEVs in neural repair strategies.

In a previous study (Nakazaki et al., 2023) demonstrated that in addition to improvements in locomotor function, intravenous delivery of MSC-sEVs exerts systemic effects on metabolic pathways affecting body growth in SCI rats (Nakazaki et al., 2023). Here we found that although the extent of neurologic improvement (motor function) was dependent on dosing parameters with more prolonged dosing having a greater effect on recovery, the improvement in body growth was the same for all dosing parameters tested. We previously demonstrated that MSC-sEV treatment resulted in a reduction in systemic serum levels of the pro-inflammatory cytokines, such as tumor necrosis factor-α (TNF-α) and interleukin-6 (IL-6), with increases in insulin-like growth factor-1 and growth hormone receptors in the liver (Nakazaki et al., 2023). From these data we proposed that infusion of MSC-sEVs reduces pro-inflammatory cytokines, leading to increased growth-related hormones and receptors in the liver which contributes to the normalization of body growth that is depressed in the SCI rats. Thus, post-injury restoration of body growth may be controlled by different signaling pathways than those which are more directly related to functional motor recovery and therefore not be as dependent on dosing parameters.

Although biodistribution data specific to the continuous infusion protocol were not obtained in this study, our previous studies demonstrated that DiR-labeled MSC-sEVs administered via daily IV injections selectively accumulated at the SCI lesion site and were preferentially internalized by CD206-positive M2 macrophages [33,39]. In the current study, we confirmed that the osmotic pump delivery system effectively delivers solutions intravenously by demonstrating continuous delivery of Evans Blue dye via the pump (Supplementary Fig. 1). Given that the same sEVs are delivered into the same venous compartment, albeit at a slower continuous rate rather than as bolus doses, we expect that the biodistribution pattern would be comparable. However, direct verification of hMSC-sEV biodistribution under continuous infusion conditions remains to be confirmed.

Taken together, the results of this study argue that continuous infusion over several days is the most efficacious delivery protocol and provides the most efficient use of hMSC-sEVs for therapeutic treatments. Our analyses also suggest that microRNA cargoes in hMSC-sEVs may contribute to downregulation of fibrosis pathways and inhibition of ECM deposition in a way that may be more conducive to increased axonal regeneration and restoration of neural networks. Since ongoing inflammation leads to fibrosis which impedes axonal sprouting and regeneration, prolonged continuous application of hMSC-sEVs may be necessary to more effectively stabilize the inflammatory response and reduce ECM deposition at the injury site. Thus, the temporal distribution of dosing may be a critical factor in determining the efficacy of hMSC-sEVs based therapy for spinal cord injury. These results have important implications for designing protocols for hMSC-sEVs treatments of SCI in order to optimize their therapeutic benefit and suggest that MSC-sEVs modulation of fibrosis pathways may be a key component to their mechanisms of action.

## Author contributions

M.N., K.L.L., M.T., and J.D.K. designed the study. M.N., K.L.L., and M.T. performed the experiments; M.T. was responsible for the preparation of extracellular vesicles. T.S.S. provided advice on FACS analysis. Y.T. provided advice on RNA-seq, microRNA-seq, and pathway analysis. M.N., K.L.L., M.T. and J.D.K. analyzed the data. M.N., K.L.L. and M.T. drafted the manuscript. Y.T., T.S.S. and J.D.K. provided critical revisions. All authors reviewed the manuscript.

## Data availability

The data that support the findings of this study are available from the corresponding author upon reasonable request

## Declaration of competing interest

The authors declare the following financial interests/personal relationships which may be considered as potential competing interests: Jeffery Kocsis reports financial support was provided by RR&D and BLR&D Services of U.S. Department of Veterans Affairs. If there are other authors, they declare that they have no known competing financial interests or personal relationships that could have appeared to influence the work reported in this paper.
