# Supplementary material for: Continuous intravenous infusion of human mesenchymal stromal cell-derived small extracellular vesicles in spinal cord injured rat modulates extracellular matrix and has greater therapeutic efficacy than multiple single injections
Source: Neurotherapeutics. 2026 Mar 26;23(3):e00892. doi: 10.1016/j.neurot.2026.e00892 (PMC13054005; doi:10.1016/j.neurot.2026.e00892)
Supplement: Multimedia component 1 [file mmc1.docx]

Continuous intravenous infusion of human mesenchymal stromal cell-derived small extracellular vesicles in spinal cord injured rat modulates extracellular matrix and has greater therapeutic efficacy than multiple single injections

Masahito Nakazaki^1,2,3^, Karen L. Lankford^1,2^, Masayuki Toyoshima^1,2,4^,

Yoshiaki Tanaka^5^, Tomokazu S. Sumida^6^ and Jeffery D. Kocsis^1,2^

^1^Department of Neurology, Yale University School of Medicine, New Haven, Connecticut, 06510, USA;^2^Center for Neuroscience and Regeneration Research, VA Connecticut Healthcare System, West Haven, Connecticut, 06516, USA; ^3^Department of Neural Regenerative Medicine, Research Institute for Frontier Medicine, Sapporo Medical University School of Medicine, Sapporo, Hokkaido, 060-8556, Japan; ^4^, Division of Regenerative and Advanced Therapy, Nipro Corporation, Osaka, Osaka, 531-8510, Japan; ^5^, Maisonneuve-Rosemont Hospital Research Center (CRHMR), Department of Medicine, University of Montreal, Quebec, Canada; ^6^ Departments of Neurology, Yale University School of Medicine, New Haven, CT, USA

Correspondence: Jeffery D. Kocsis, Ph.D.

Yale University School of Medicine

Neuroscience Research Center (127A)

VA Connecticut Health Care System

West Haven, Connecticut 06516

Tel: (203) 937-3802

FAX: (203) 937-3801

E-mail: [jeffery.kocsis@yale.edu](mailto:jeffery.kocsis@yale.edu)


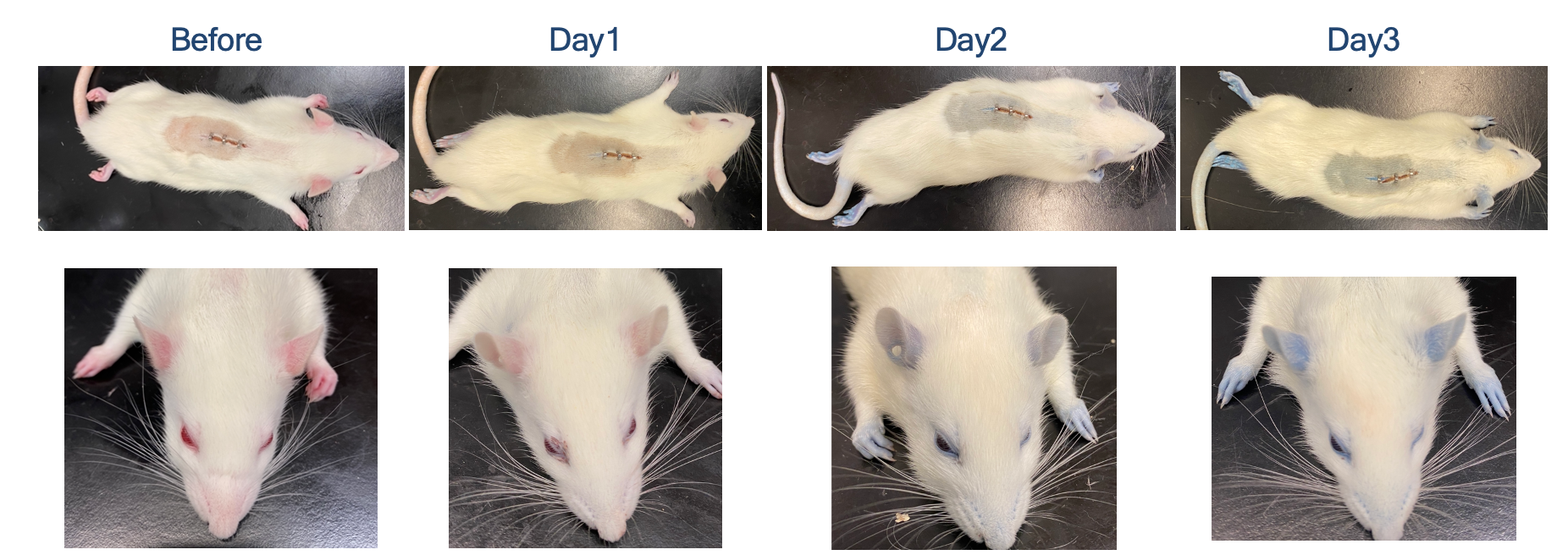
 **Supplementary Figure 1**

**Supplementary Figure 1: Gradual infusion of Evans Blue in SCI rat via osmotic pump**

Upper and lower panels show whole-body (upper) and head-and-forequarters (lower) images of a spinal cord injury (SCI) rat. Images were captured before and 1, 2, and 3 days after the implantation of an osmotic pump delivering 4% Evans Blue dye in saline. The increasing blue discoloration over time indicates a steady, controlled release of the dye, demonstrating consistent delivery over a 3-day period. SCI: Spinal Cord Injury, Evans Blue: A dye used to trace vascular permeability or fluid distribution


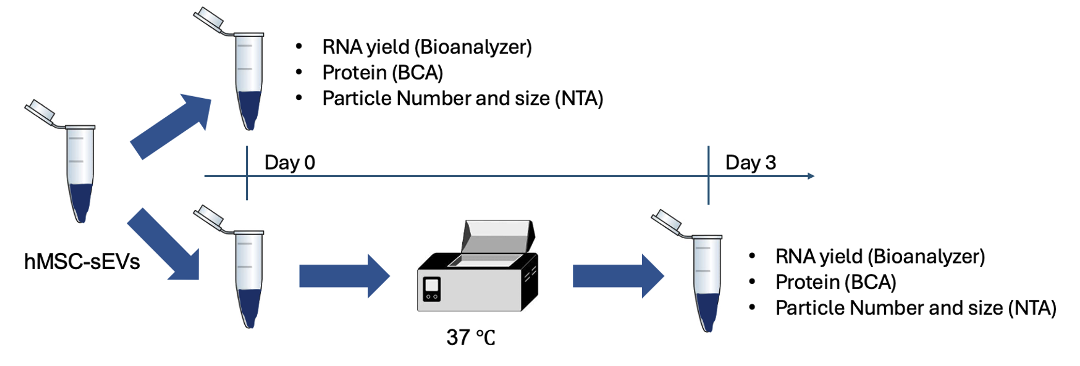
**Supplementary Figure 2**

Supplementary Figure 2: Schematic of the experimental design to evaluate stability of hMSC-sEVs after incubation at 37°C for 3 days.

hMSC-sEV preparations stored at −80°C were thawed and aliquoted into two groups. One aliquot was analyzed immediately (Day 0) and the other was incubated at 37°C for 3 days (Day 3) to simulate the maximum duration within the osmotic pump reservoir before replacement. At each timepoint, samples were assessed for RNA yield (Agilent Bioanalyzer, see (b)), total protein content (BCA assay), and particle size distribution and concentration (nanoparticle tracking analysis, NTA). Detailed methodologies are described in MATERIALS AND METHODS.

**Supplementary Figure 3**


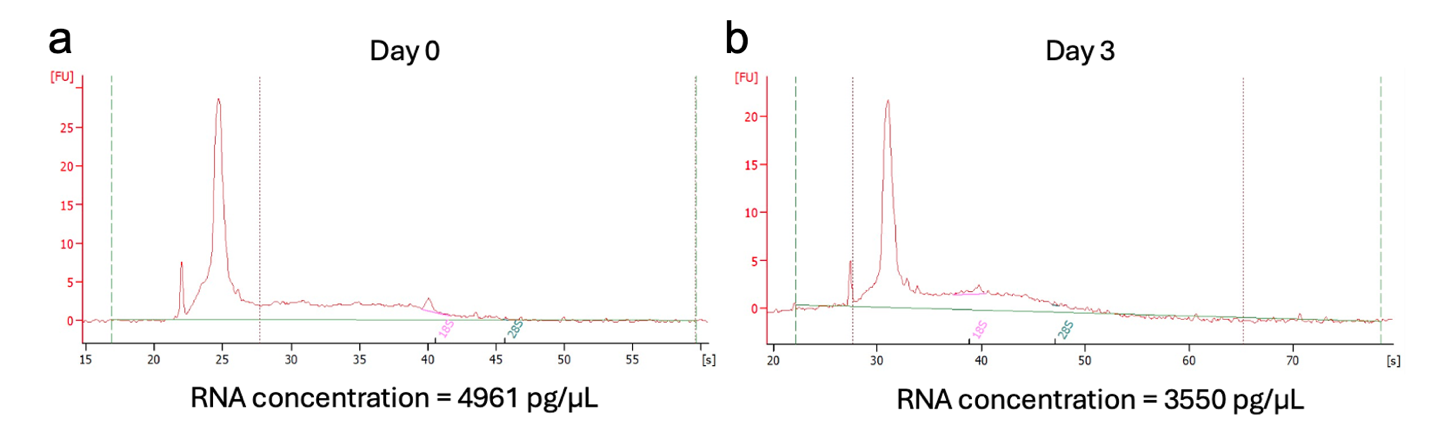


Supplementary Figure 3: RNA integrity of hMSC-sEVs after incubation at 37°C for 3 days Representative electropherograms from the Agilent 2100 Bioanalyzer using the RNA 6000 Pico Kit (Agilent Technologies, Santa Clara, CA, USA) at Day 0 (a) and Day 3 (b). Total RNA was extracted from hMSC-sEV samples using the miRNeasy Serum/Plasma Kit (QIAGEN) following the manufacturer's protocol. Extracted RNA (1 μL) was loaded onto RNA 6000 Pico chips according to the manufacturer's instructions. The RNA 6000 Pico assay employs microfluidic electrophoretic separation with fluorescence detection optimized for low-concentration RNA samples (sensitivity range: 50–5000 pg/μL). The electropherograms display size-based separation of RNA species, with the dominant peak at approximately 25 seconds representing the small RNA fraction characteristic of sEVs. RNA concentrations decreased from 4961 pg/μL at Day 0 to 3550 pg/μL at Day 3, corresponding to a 28.4% reduction. Despite this loss, the overall electropherogram profile, including the small RNA peak distribution, was preserved, indicating that the RNA cargo within hMSC-sEVs remained largely intact after 3 days at 37°C.

**Supplementary Figure 4**


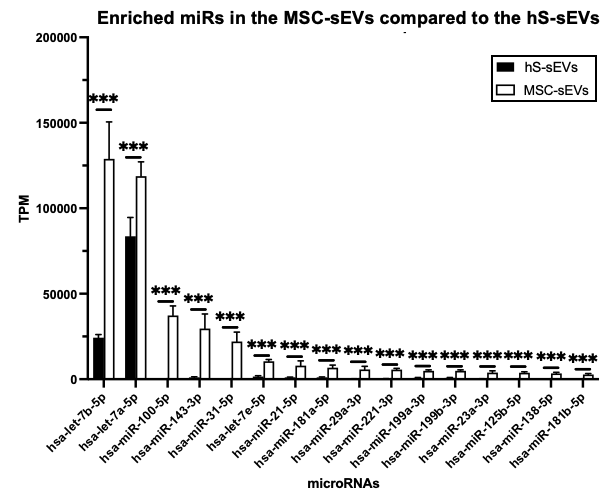


**Supplementary Figure 4: Relatively abundant microRNAs in hMSC-sEVs compared to hS-sEVs**

Bar graph illustrating the levels of enriched microRNAs (miRNAs) in mesenchymal stromal/stem cell-derived small extracellular vesicles (hMSC-sEVs) compared to human serum-derived small extracellular vesicles (hS-sEVs). The miRNA expression was measured using next-generation sequencing. The top 15 significantly enriched miRs in hMSC-sEVs are shown, with notable increases in hsa-let-7b-5p, hsa-miR-100-5p, and hsa-miR-143-3p, among others. Data are represented as transcripts per million (TPM), with significant differences marked as ***p < 0.001. hMSC-sEVs: Human Mesenchymal Stromal/Stem Cell-Derived Small Extracellular Vesicles, hS-sEVs: Human Serum-Derived Small Extracellular Vesicles, miRNA (miR): MicroRNA, TPM: Transcripts Per Million, NGS: Next-Generation Sequencing.


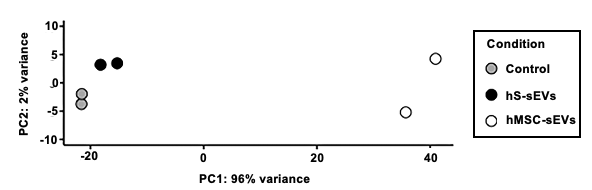
**Supplementary Figure 5**

**Supplementary Figure 5: Principal Component Analysis (PCA) of gene expression in macrophages treated with MSC-sEVs or hS-sEVs.**
Principal Component Analysis (PCA) plot displaying the variance in gene expression between control (CTR, no-EV treatment) macrophages, macrophages treated with human serum-derived small extracellular vesicles (hS-sEVs), and macrophages treated with human mesenchymal stromal/stem cell-derived small extracellular vesicles (hMSC-sEVs). The first principal component (PC1), accounting for 96% of the variance, shows a significant separation between macrophages treated with hMSC-sEVs (open circles) and both the CTR (grey circles) and hS-sEVs (black circles) groups. This indicates that MSC-sEV treatment induces substantial changes in mRNA expression profiles compared to the hS-sEVs and control groups. PCA: Principal Component Analysis, CTR: Control, EV: Extracellular Vesicle, hS-sEVs: Human Serum-Derived Small Extracellular Vesicles, hMSC-sEVs: Human Mesenchymal Stromal/Stem Cell-Derived Small Extracellular Vesicles, PC1: Principal Component 1, mRNA: Messenger Ribonucleic Acid.

| Primary Antibody (Manufacture, catalog #, dilution used) | Second Antibody (Manufacture, catalog #, dilution used) | Application |
| --- | --- | --- |
|  |  |  |
| Exosome specific protein markers |  |  |
| rabbit monoclonal anti-CD 9 antibody  (Abcam, ab92726, 1:1000) | Goat Anti-Rabbit IgG (H + L)-HRP Conjugate (BIO-RAD, 1706515, 1:25000) | Western blot analysis in Fig.1(c) |
| rabbit polyclonal anti-CD 63 antibody  (LS-Bio, LS-C408817, 1:1000) | Goat Anti-Rabbit IgG (H + L)-HRP Conjugate (BIO-RAD, 1706515, 1:25000) | Western blot analysis in Fig.1(c) |
| rabbit polyclonal anti-Alix antibody  (Proteintech,12422-1-AP, 1:1000) | Goat Anti-Rabbit IgG (H + L)-HRP Conjugate (BIO-RAD, 1706515, 1:25000) | Western blot analysis in Fig.1(c) |
| rabbit polyclonal anti-Calnexin antibody (Abcam, ab22595, 1:1000) | Goat Anti-Rabbit IgG (H + L)-HRP Conjugate (BIO-RAD, 1706515, 1:25000) | Western blot analysis in Fig.1(c) |
|  |  |  |
| Extracellular matrix markers |  |  |
| rabbit polyclonal anti-fibronectin antibody  (Sigma-Aldrich, F3648, 1:1000) | Goat Anti-Rabbit IgG (H + L)-HRP Conjugate (BIO-RAD, 1706515, 1:25000) | Western blot analysis in Fig. 7 (h)-(i) |
| rabbit polyclonal anti-fibronectin antibody  (Sigma-Aldrich, F3648, 1:400) | Alexa Fluor 488-conjugated donkey anti-rabbit (Thermofisher, A21206, 1:1000) | Immunofluorescent fibronectin marker in Fig. 7 (a)-(g) |
| Rabbit recombinant multiclonal anti-Collagen I antibody  (Abcam, ab316222, 1:1000) | Goat Anti-Rabbit IgG (H + L)-HRP Conjugate (BIO-RAD, 1706515, 1:25000) | Western blot analysis in Fig. 7 (h)-(i) |
| Rabbit recombinant multiclonal anti-Collagen I antibody  (Abcam, ab316222, 1:500) | Alexa Fluor 488-conjugated donkey anti-rabbit (Thermofisher, A21206, 1:1000) | Immunofluorescent Collagen I marker in Fig. 7 (a)-(g) |
| Rabbit recombinant monoclonal anti-Collagen Ⅴ antibody  (Abcam, ab275881, 1:1000) | Goat Anti-Rabbit IgG (H + L)-HRP Conjugate (BIO-RAD, 1706515, 1:25000) | Western blot analysis Fig. 7 (h)-(i) |
|  |  |  |
| Cell sorting |  |  |
| BV421 Mouse Anti-Rat CD45  (BD Bioscience, 740042, 1:50) | - | Fluorescence-Activated Cell Sorting, viability marker in Fig 5(b) |
| FITC Mouse Anti-Rat CD11b antibody \| OX-42 (Bio-Rad, MCA275FT, 1:10) | - | Fluorescence-Activated Cell Sorting, viability marker in Fig 5(b) |
| 7-AAD Viability Staining Solution  (Biolegend, 420403, 5 µl per million cells) | - | Fluorescence-Activated Cell Sorting, viability marker in Fig 5(b) |
|  |  |  |
| Others |  |  |
| goat polyclonal anti-CD 206 antibody  (R&D Systems, AF2535, 1:50) | Alexa Fluor 594-conjugated donkey anti-goat (Thermofisher, A11058, 1:1000) | Immunofluorescent M2 macrophages marker in Fig. 7 (a)-(g) |
| rabbit monoclonal anti-GAPDH antibody  (Cell Signaling, 2118s, 1:20000) | Goat Anti-Rabbit IgG (H + L)-HRP Conjugate (BIO-RAD, 1706515, 1:25000) | Used as the inner control in Western blot analysis |

**Supplementary Table 1**

**Supplementary Video 1. Representative videos of locomotor function at 70 days post-SCI.**

Videos show the open-field locomotor function of representative rats at 70 days post-injury. Treatments were initiated at 7 days post-SCI and administered for either 3 days (a-d) or 6 days (e-h).

**Supplementary Video 1a**

A representative rat treated with daily injections of PBS for 3 days.

**Supplementary Video 1b**

A representative rat treated with daily injections of hMSC-sEVs for 3 days.

**Supplementary Video 1c**

A representative rat treated with continuous infusion of PBS via an osmotic pump for 3 days.

**Supplementary Video 1d**

A representative rat treated with continuous infusion of hMSC-sEVs via an osmotic pump for 3 days.

**Supplementary Video 1e**

A representative rat treated with daily injections of PBS for 6 days, showing minimal hindlimb weight support and tail dragging.

**Supplementary Video 1f**

A representative rat treated with daily injections of hMSC-sEVs for 6 days, showing some weight-supported steps.

**Supplementary Video 1g**

A representative rat treated with continuous infusion of PBS via an osmotic pump for 6 days, demonstrating severe motor deficits.

**Supplementary Video 1h**

A representative rat treated with continuous infusion of hMSC-sEVs via an osmotic pump for 6 days, showing consistent weight-supported plantar stepping and coordinated movement.
